# The prognostic significance of anti-angiogenesis therapy in ovarian cancer: a meta-analysis

**DOI:** 10.1186/s13048-015-0181-z

**Published:** 2015-08-06

**Authors:** Jun Li, Shufen Li, Ruifang Chen, Hailin Yu, Xin Lu

**Affiliations:** Obstetrics and Gynecology Hospital, Fudan University, Shanghai, 200011 China; Department of Obstetrics and Gynecology of Shanghai Medical College, Fudan University, Shanghai, 200032 China; Shanghai Key Laboratory of Female Reproductive Endocrine Related Diseases, Shanghai, 200011 China; Department of Biochemistry and Molecular Biology, Key Laboratory of Molecular Medicine, Ministry of Education, Fudan University Shanghai Medical College, Shanghai, 200032 China; Present address: Department of Gynecology, Obstetrics and Gynecology Hospital of Fudan University, No.419, Fangxie Road, Shanghai, 200011 China

**Keywords:** Ovarian cancer, Anti-angiogenesis therapy, Prognosis, Meta-analysis

## Abstract

**Objective:**

The prognostic value of anti-angiogenesis therapy in ovarian cancer patients is currently under debate. In this study, we assessed the effects of anti-angiogenesis therapy on the progression free survival (PFS) and overall survival (OS) of ovarian cancer patients.

**Materials and methods:**

PubMed was searched to identify relevant studies that evaluated the therapeutic value of anti-angiogenic agents in ovarian cancer (the final search was current to Dec. 13th 2014). Reviews of each study were conducted, and the data were extracted. The primary outcomes that were analysed were progression free survival (PFS) and overall survival (OS). The pooled hazard ratio (HR) and 95 % confidence intervals (CIs) were calculated using the random and fixed-effects models, and subgroup and sensitivity analyses were subsequently performed.

**Results:**

A total of 12 studies were included in the meta-analysis. The overall analysis revealed that the incorporation of anti-angiogenesis therapy was significantly associated with a longer PFS (HR, 0.66; 95 % CI, 0.58-0.75; *P* < 0.01) and a longer OS (HR, 0.89; 95 % CI, 0.82-0.97; *P* = 0.01) in the total population, and these findings were confirmed by one-way sensitivity analyses. Further subgroup analyses demonstrated that the administrations of each of the agents were associated with improved PFSs. The prognostic value of anti-angiogenesis therapy for the OS was significant in the trebananib subgroup (HR, 0.81; 95 % CI, 0.67-0.99; *P* = 0.04). The bevacizumab subgroup exhibited a similar trend that did not reach statistical significance (HR, 0.90; 95 % CI, 0.80-1.01; *P* = 0.08).

**Conclusions:**

The present meta-analysis indicated that anti-angiogenesis therapy in ovarian cancer patients was associated with a better clinical outcome. Further studies are warranted to identify the specific subgroup of patients who are most likely to benefit from anti-angiogenesis therapy.

## Background

Worldwide, approximately 238,000 women are diagnosed with ovarian cancer and 151,000 women succumb to this disease in 2012. Currently, the standard treatment for ovarian cancer is aggressive cytoreductive surgery, followed by a platinum and taxane combination chemotherapy [1]. Although 70 % of the patients will experience a complete clinical remission after the initial therapy, the majority will eventually experience a cancer progression and succumb to their disease [2]. Thus, ovarian cancer patients usually require further treatments. With the aim of improving the prognoses of ovarian cancer patients, multiple clinical trials have been conducted to explore new therapies, including anti-angiogenesis therapy [3].

Angiogenesis is a complex, multi-step process that is controlled by several key pathways, including the vascular endothelial growth factor (VEGF) pathway, the platelet-derived growth factor (PDGF) pathway, the fibroblast growth factor (FGF) pathway, and the angiopoietin-Tie2 receptor pathway [4]. Accumulating evidence has demonstrated that the disruption of the angiogenesis axis is involved in the progression of ovarian cancer *via* the promotion of tumor growth, ascites, and metastases [5]. Due to these findings, anti-angiogenesis therapy has been extensively studied in patients with ovarian cancer [6, 7]. Promisingly, extended progression free survival (PFS) has been observed in patients who receive anti-angiogenesis therapy. However, it is not appropriate to simplify the clinical benefit of new drugs to improvements in PFS, and overall survival (OS) is also a key secondary study endpoint. Moreover, the OS benefit of anti-angiogenesis therapy remains controversial. To acquire improved better insight into the clinical benefits of anti-angiogenesis therapy for ovarian cancer, we performed a meta-analysis of the published literature on this topic. Specifically, we assessed the prognostic value of anti-angiogenesis therapy for the PFS and OS of ovarian cancer patients.

## Methods

We designed, analysed, and reported our meta-analysis according to the PRISMA Statement guidelines.

### Search strategy

A literature search (the final search was current to Dec. 13th, 2014) of PubMed for articles addressing the clinical benefit of anti-angiogenesis therapy in ovarian cancer was performed using the following keywords: (bevacizumab OR trebananib OR pazopanib OR cediranib OR nintedanib OR imatinib OR perifosine OR sorafenib OR sunitinib OR AEE788 OR aflibercept OR “AMG 386” OR “BIBF 1120”) AND (“ovarian cancer” OR “ovarian neoplasm” OR “ovarian tumor” OR “ovarian carcinoma”). The results were limited to peer-reviewed, English language reports. Abstracts from ASCO, ESMO and ESGO were also screened to identify the potentially relevant studies. Additionally, the references lists of the retrieved articles were examined for potentially eligible studies.

### Eligibility criteria

The studies were deemed eligible for inclusion in the meta-analysis based on the following criteria: (i) either the PFS or the OS benefit of anti-angiogenesis therapy in ovarian cancer patients was reported or could be extrapolated from reported data, and (ii) only randomized clinical trials were included. Additionally, we scrutinized all of included articles to avoid the potential influence of redundant studies.

### Data extraction

The data extracted for this meta-analysis included the first author, anti-angiogenic agents, journal, phase, year, treatment settings, and hazard ratios (HRs) for the PFS and OS.

### Statistical analysis

Briefly, the HRs and their respective 95 % CI were used to assess the prognostic effect of anti-angiogenesis therapy for ovarian cancer patients. The pooled HRs for the PFS and OS were evaluated with fixed or random-effects models. The potential heterogeneity between studies was estimated using the Cochran’s Q-test and the I^2^ index. A fixed-effects model was used employed when the I^2^ ≤ 50 %; otherwise, a random-effects model was used. Publication bias was assessed with Egger’s and Begg’s test. Additionally, one-way sensitivity analyses were performed to evaluate the effects of the individual studies by estimating the average HRs in the absence of each study. All analyses were performed with STATA 11.0 software.

## Results

### Identification and characteristics of relevant studies

**Fig. 1:**
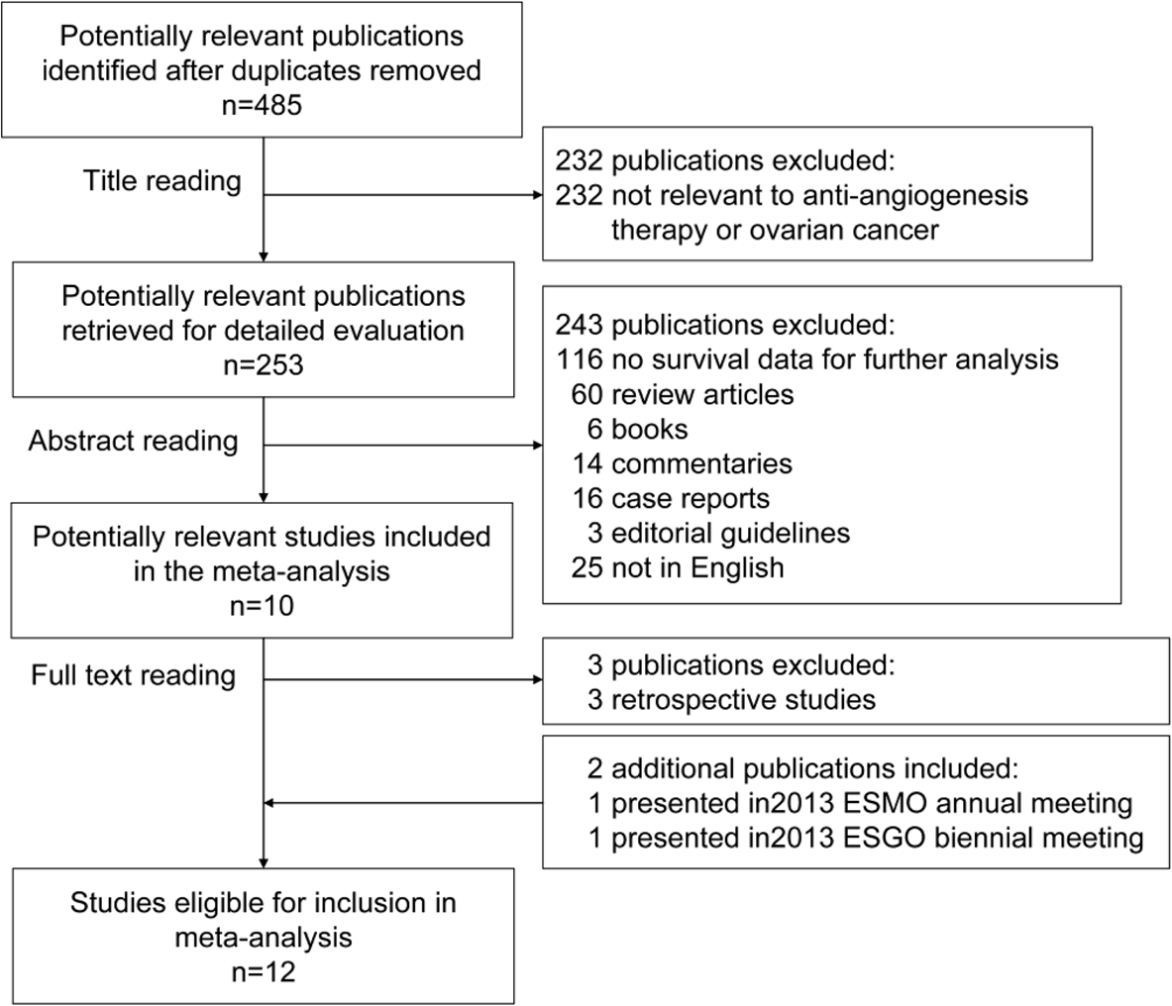
Flow chart of the search strategy used for the selection of eligible studies

We screened 485 potentially relevant articles in our systematic literature search and identified 10 eligible studies (Fig. 1). Two additional reports presented at the 2013 ESMO meeting and the 2013 ESGO biennial meeting were also included in our meta-analysis (Fig. 1). The characteristics of the included studies are shown in Table 1 [8–19].

**Table 1:** Characteristics of included studies

First author	Agents	Journal	Phase	Year	Treatment setting	No. of patients		Median follow-up time (months)		FIGO stage	Hazard ratio (95 % CI)	
						Control group	Anti-angiogenic group	Control group	Anti-angiogenic group		PFS	OS
Pujade-Lauraine E [8]	Bevacizumab	JCO	III	2014	Recurrent	182	179	13.9	13	—	0.48 (0.38–0.60)	0.85 (0.66–1.08)
Aghajanian C [9]	Bevacizumab	JCO	III	2012	Recurrent	242	242	24	24	—	0.48 (0.39–0.61)	1.03 (0.79–1.33)
Perren TJ [10]	Bevacizumab	NEJM	III	2011	Primary	764	764	19.4	19.4	I-IV	0.87 (0.77–0.99)	0.85 (0.69–1.04)
Burger RA [11]	Bevacizumab	NEJM	III	2011	Primary	625	623	17.4	17.4	III-IV	0.64 (0.55–0.76)	0.92 (0.73–1.15)
Monk BJ [12]	Trebananib	Lancet Oncology	III	2014	Recurrent	458	461	10.1	10.1	—	0.66 (0.57–0.77)	0.86 (0.69–1.08)
Karlan BY [13]^a^	Trebananib	JCO	II	2012	Recurrent	55	53	14.9	15.4	I-IV	0.81 (0.51–1.30)	0.60 (0.34–1.06)
Karlan BY [13]^b^	Trebananib	JCO	II	2012	Recurrent	55	53	14.9	15.2	I-IV	0.75 (0.49–1.21)	0.77 (0.45–1.31)
du Bois A [14]	Pazopanib	JCO	III	2014	Primary	468	472	24	24	II-IV	0.77 (0.64–0.91)	1.08 (0.87–1.33)
Liu JF [15]	Cediranib	Lancet Oncology	II	2014	Recurrent	46	44	16.6	16.6	—	0.42 (0.23–0.76)	—
Ledermann JA [16]	Cediranib	2013 ESMO meeting	III	2013	Recurrent	—	—	—	—	—	0.57 (0.45–0.74)	0.70 (0.51–0.99)
Ledermann JA [17]	Nintedanib	JCO	II	2011	Recurrent	40	43	—	—	I-IV	0.65 (0.41–1.02)	0.84 (0.51–1.39)
du Bois A [18]	Nintedanib	2013 ESGO meeting	III	2013	Primary	455	911	—	—	IIB-IV	0.84 (0.72–0.98)	—
Gotlieb WH [19]	Aflibercept	Lancet Oncology	II	2012	Recurrent	26	29	—	—	—	—	1.01 (0.56–1.86)

^a^Trebananib:10mg/kg; ^b^ Trebananib:3mg/kg

### The effects of anti-angiogenesis therapy on PFS and OS

**Fig. 2:**
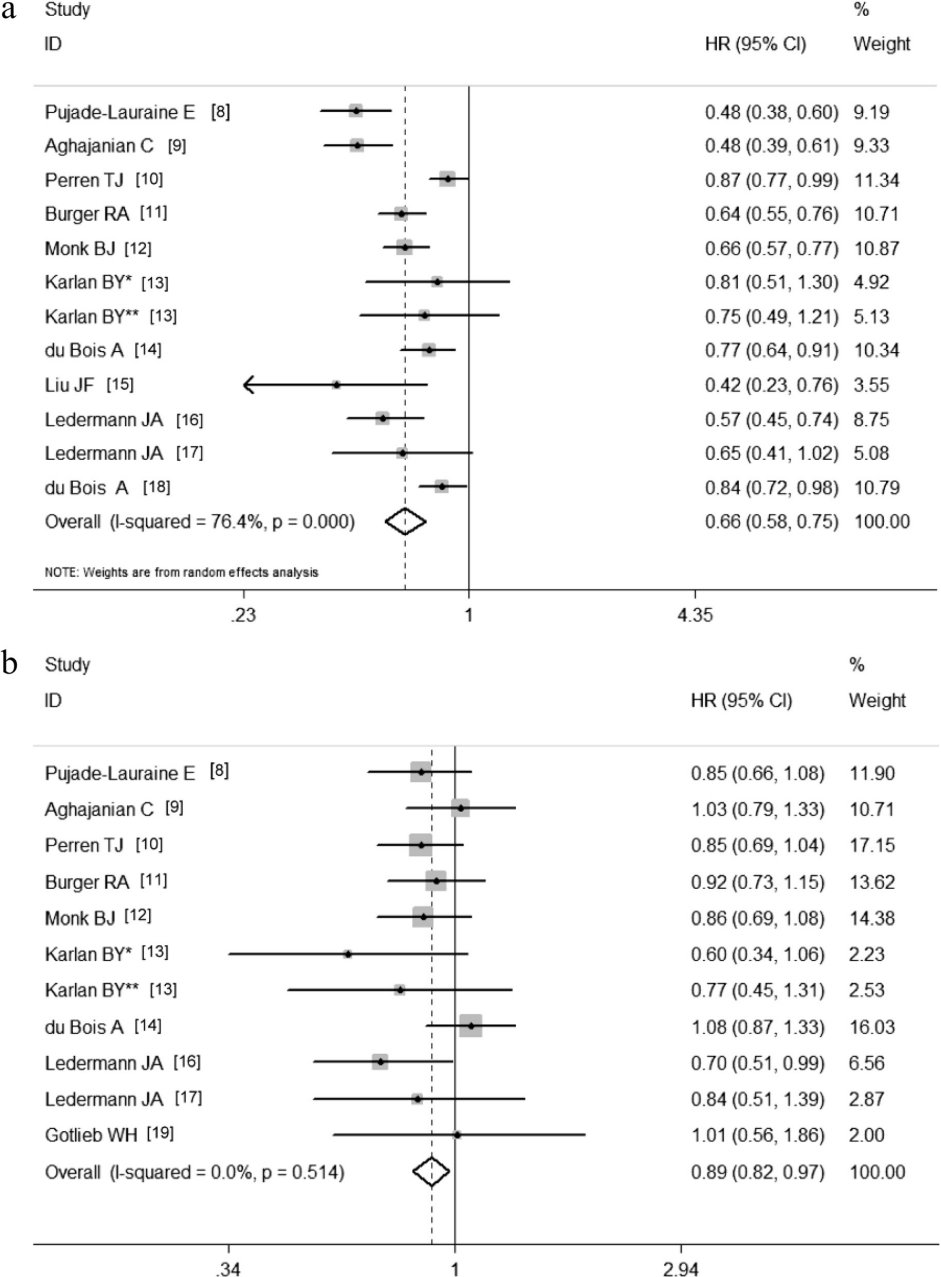
Forest plots of the clinical benefit of anti-angiogenesis therapy in ovarian cancer. **a** The effects of anti-angiogenic agents on PFS; **b** The effects of anti-angiogenic agents on OS

The PFS HRs were available in 11 studies. The estimated pooled HR revealed that the incorporation of anti-angiogenesis therapy was associated with an improved PFS in ovarian cancer patients (HR, 0.66; 95 % CI, 0.58-0.75; *P* < 0.01; random effects; Fig. 2a). The heterogeneity between the studies was significant (*P* = 0.00, I^2^ = 76.4 %). There was no significant publication bias (P_Begg_ = 0.30; P_Egger_ = 0.17).

The HRs for OS were available in 10 studies. The estimated pooled HR showed that anti-angiogenesis therapy contributed to improved OS in ovarian cancer patients (HR, 0.89; 95 % CI, 0.82-0.97; *P* = 0.01; fixed effects; Fig. 2b). No significant heterogeneity was detected among studies (*P* = 0.514, I^2^ = 0.0 %). There was no significant publication bias (P_Begg_ = 0.53; P_Egger_ = 0.21).

**Fig. 3:**
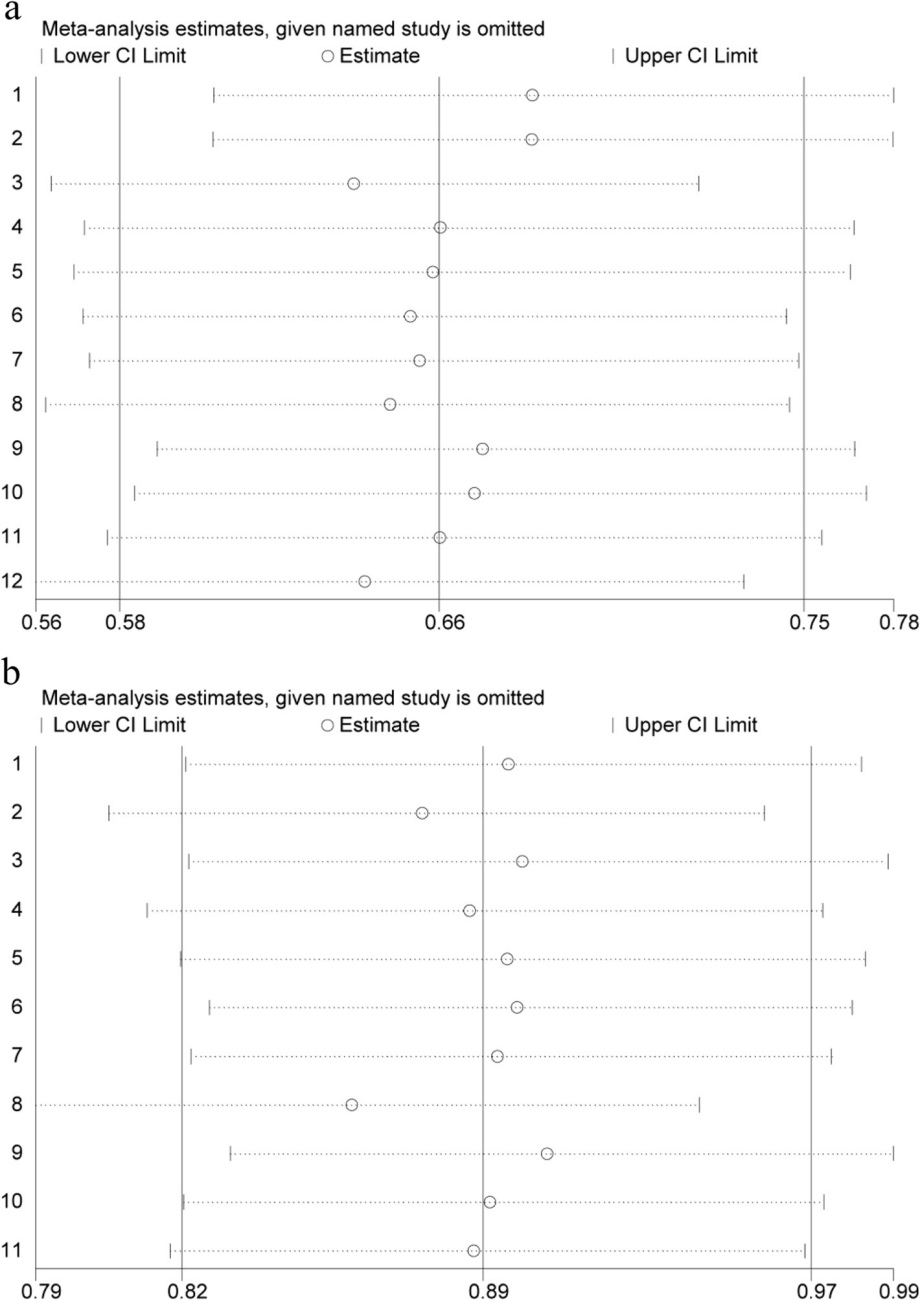
Confirmation of the stability of our results via one-way sensitivity analyses. **a**, **b** One-way sensitivity analyses confirmed the effects of anti-angiogenesis therapy on PFS (**a**) and OS (**b**)

Our results were further confirmed by one-way sensitivity analysis (Fig. 3a, b).

### Subgroup analyses of anti-angiogenesis in ovarian cancer

**Fig. 4:**
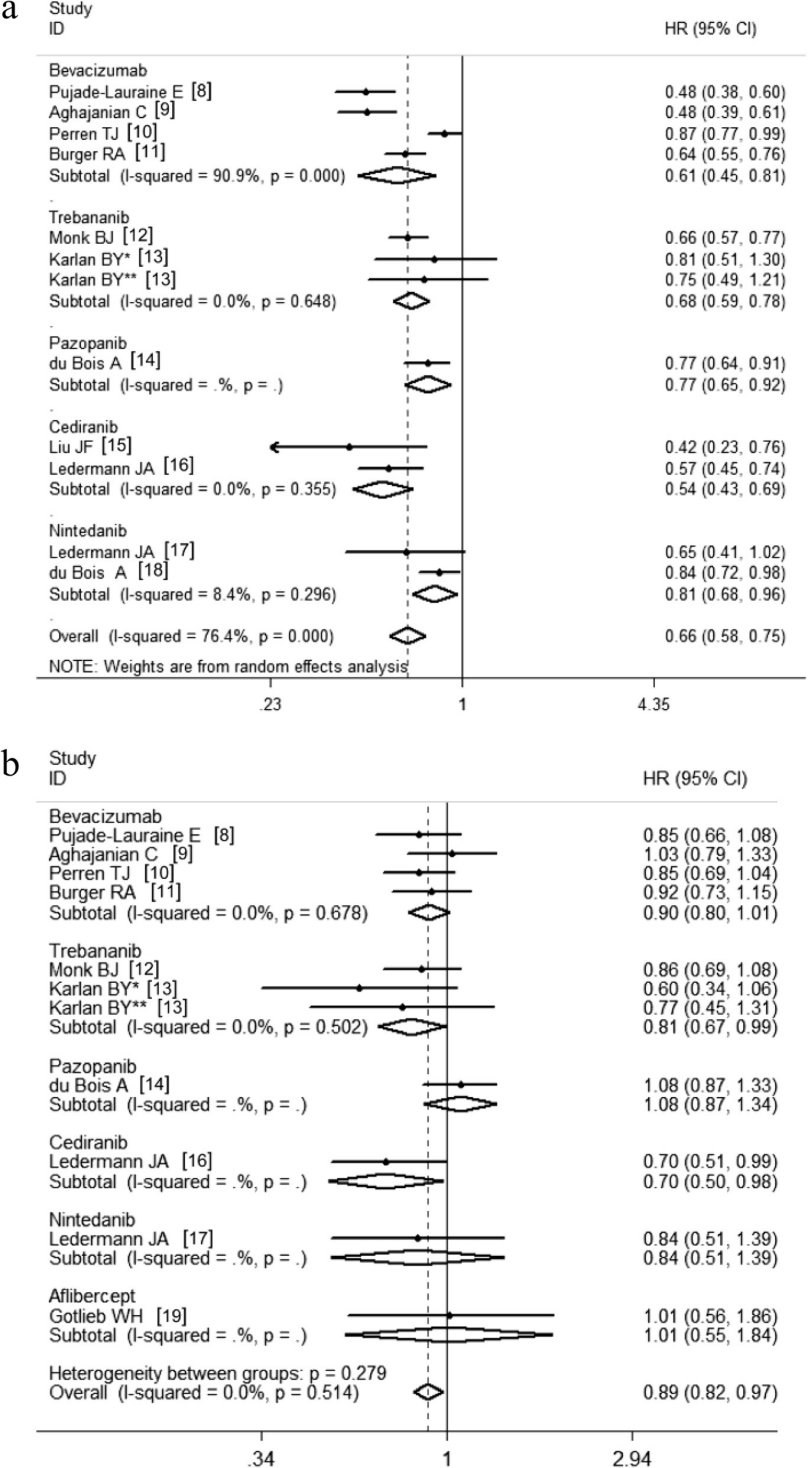
Subgroup analyses stratified according to anti-angiogenic agents. **a** The effects of anti-angiogenic agents on PFS; (**b**) The effects of anti-angiogenic agents on OS

Subgroup analyses stratified by the anti-angiogenesis agents indicated that administrations of each of the agents were associated with improved PFS (Fig. 4a). The prognostic value of anti-angiogenesis therapy for OS was significant in the trebananib subgroup (HR, 0.81; 95 % CI, 0.67-0.99; *P* = 0.04; fixed effects; Fig. 4b). The bevacizumab subgroup exhibited a similar trend that did not reach statistical significance (HR, 0.90; 95 % CI, 0.80-1.01; *P* = 0.08; fixed effects; Fig. 4b).

**Fig. 5:**
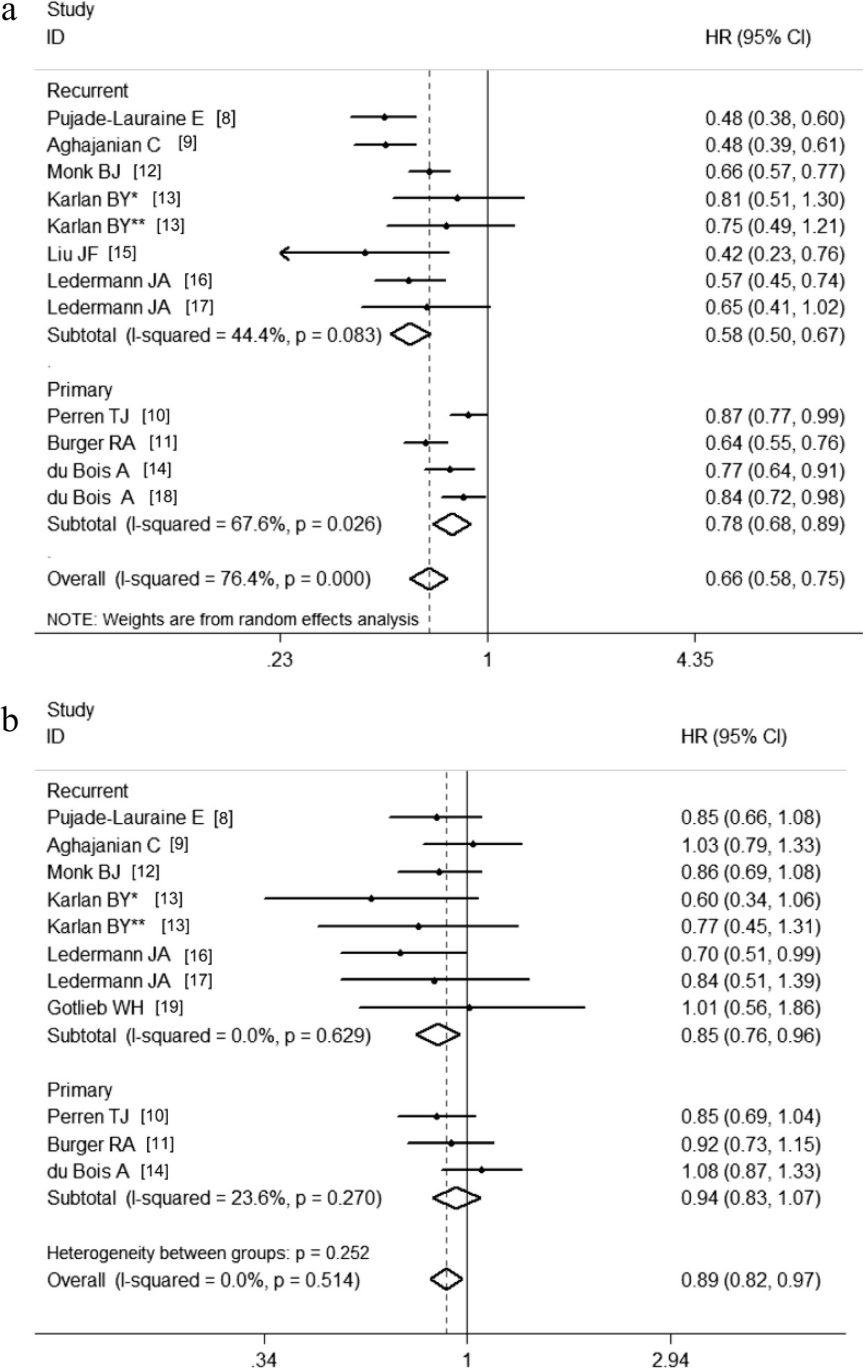
Subgroup analyses stratified according to treatment settings. **a** The effects of anti-angiogenesis therapy of PFS stratified according to treatment setting; **b** The effects of anti-angiogenesis therapy on OS according to treatment settings

Moreover, the clinical benefit of anti-angiogenesis therapy differed between the patients with primary ovarian cancer and the patients with recurrent ovarian cancer. Specifically, improved PFS was observed in both recurrent setting (HR, 0.58; 95 % CI, 0.50-0.67; *P* = 0.02; random effects; Fig. 5a) and primary setting (HR, 0.78; 95 % CI, 0.68-0.89; *P* = 0.01; random effects; Fig. 5a). However, the prognostic value of anti-angiogenesis therapy for OS was significant in the patients with recurrent ovarian cancer (HR, 0.85; 95 % CI, 0.76-0.96; *P* = 0.01; fixed effects; Fig. 5b), but not in the primary setting (HR, 0.94; 95 % CI, 0.83-1.07; *P* = 0.35; fixed effects; Fig. 5b).

## Discussion

Angiogenesis has been implicated in the pathogenesis and progression of ovarian cancer potentially due to the promotion of tumor growth, ascites, and metastases [5]. Therefore, therapies based on angiogenesis-specific pathway are being extensively studied in ovarian cancer [6, 7]. Our present meta-analysis was based on a pool of 8 phase III and 4 phase II clinical trials and thus differed from the two existing meta-analyses, which included only clinical trials involving bevacizumab [20, 21]. Namely, these two meta-analyses indicated that the addition of bevacizumanb to chemotherapy led to significant improvement in PFS and also increased the occurrence of adverse events including gastrointestinal events, hypertension, proteinuria, and aterial thromboembolism [20, 21]. The present meta-analysis revealed that the incorporation of anti-angiogenesis therapy was significantly associated with improvements in PFS and OS of ovarian cancer patients. The pooled findings were further confirmed by one-way sensitivity analyses. More interestingly, the subgroup analyses revealed that the patients with recurrent ovarian cancer derived greater OS benefit from the anti-angiogenesis agents. In contrast, anti-angiogenesis therapy in the primary setting conferred no significant OS benefit to ovarian cancer patients. These pooled results do not indicate that the recurrence setting is ideal for the incorporation of anti-angiogenesis into the treatment of ovarian cancer. In the front-line setting, although the ICON7 and GOG218 trial failed to identify significant differences in the OS benefits for patients according to whether bevacizumab was added to the treatment across the entire populations of those studies, the addition of bevacizumab to front line therapy does confer an OS improvement for patients who are at a high risk for progression [10, 11]. This finding raised a question regarding patient selection that that led to the use of individualized treatment regimens. Unfortunately, there is still a paucity of reliable biomarkers to predict the clinical benefit of anti-angiogenesis therapy [22]. This paucity inspired us to identify specific biomarker signatures that can be used to stratify patients with ovarian cancer according to the expected benefit of anti-angiogenesis therapy [22, 23]. Possible serum biomarkers including mesothelin, FLT4, AGP, and CA-125 were investigated [24]. Additionally, the utilities of adiposity measurements and dynamic contrast-enhanced magnetic resonance imaging (DCE-MRI) results as clinical biomarkers for anti-angiogenesis therapy are also currently under investigation [25, 26]. More recently, miR-378 and its downstream targets, ALCAM and EHD1, have been proven to be potential biomarkers of the response to anti-angiogenic therapy in ovarian cancer [27]. The challenge ahead is to validate these biomarkers and implement their use in clinical practice with the goal of providing improved guidance regarding the use of anti-angiogenic agents.

Another intriguing finding of present study is that trebananib seems to be active in the treatment of recurrent ovarian cancer. Improvements in both PFS and OS were observed in the patients who were treated with trebananib in the recurrent setting. Another phase 3 trial assessing the potential benefit of trebananib in the recurrent setting is underway (TRINOVA-2; NCT01281254). Moreover, the clinical benefit of the addition of trebananib to front-line chemotherapy is also currently under investigation in a phase 3 study (TRINOVA-3; NCT01493505). Notably, the mechanism by which trebananib blocks angiogenesis and its associated toxicity profile are distinct from those of VEGF pathway inhibitors [12]. Thus, trebananib provides a non-VEGF anti-angiogenesis option for the treatment of ovarian cancer and raises the possibility that trebananib could be combined with the VEGF pathway inhibitors, e.g., bevacizumab, in the treatment of ovarian cancer.

Certain limitations must be considered when interpreting the pooled findings. First, our meta-analysis primarily focused on the PFS and OS. Indeed, the value of the PFS for assessing the clinical benefit of new drugs for ovarian cancer has been controversial. Additionally, it is not appropriate to simplify the clinical benefits of new drugs to improvements in OS, particularly when the OS benefit of a drug is marginal, but the side effects of that drug are life threatening. Thus, appropriately designed and executed randomized trials that consider the quality of life are needed [28]. Such trials should balance the efficacy, safety, toxicity and cost. Second, significant heterogeneity was found in our study. We deduced that variability in definitions of end point, measurements, experimental design, sample size, patient characteristics, and severity of the disease, may all represent a source of heterogeneity in our meta-analysis. Publication bias is another concern. We attempted to identify all of the relevant studies, but unavoidably, some studies could still be missing. As the additional high-quality clinical trials related to anti-angiogenesis therapy that are underway are completed, further analyses can be performed to validate the trends observed here.

## Conclusions

The pooled results support the notion of a prognostic value of anti-angiogenesis therapy in ovarian cancer patients. The future challenge is to identify specific subgroups of patients who stand to benefit most to anti-angiogenesis therapy.
